# Supplementary material for: Deletion of wfs1 Impairs Oligodendrocyte Precursor Cells Dorsal Distribution and Myelination Through the wfs1-hmgcs1 Axis in Zebrafish
Source: Biology (Basel). 2026 Aug 16;15(16):1402. doi: 10.3390/biology15161402 (PMC13510151; doi:10.3390/biology15161402)
Supplement: Supplementary file 1 [file biology-15-01402-s001.zip › File S1-Transcriptome sequencing data and differentially expressed genes (DEGs) in wfs1-- zebrafish at 5 dpf/2.KEGG/wfs1KOvsWT/DOWN/src/dre04114.html]

dre04114


- dre:323883

- Down regulated genes

323883(-4.8491500403135)

- dre:323883

- Down regulated genes

323883(-4.8491500403135)

- dre:565241

- Down regulated genes

565241\_2(-6.09874532631292)

- dre:323883

- Down regulated genes

323883(-4.8491500403135)

- dre:797406

- Down regulated genes

797406\_2(-2.4994687612609)

- dre:797406

- Down regulated genes

797406\_2(-2.4994687612609)

- dre:562282

- Down regulated genes

562282\_1(-5.61712483666314)

- dre:324475

- Down regulated genes

324475\_1(-3.125210470257)

- dre:245702

- Down regulated genes

245702\_1(-1.45433245048201)

- dre:324475

- Down regulated genes

324475\_1(-3.125210470257)

Close
